# Embolic Stroke of Undetermined Source (ESUS): Exploring the Neurocardiological Axis and Its Clinical Implications

**DOI:** 10.3390/medicina61071252

**Published:** 2025-07-10

**Authors:** Gabriela Dumachita Sargu, Roxana Covali, Cristiana Filip, Tudor Butureanu, Mona Akad, Ioana Păvăleanu, Andrei Ionuț Cucu, Amelian Mădălin Bobu, Laura Riscanu, Diana Lacatusu, Madalina Irina Smihor, Radu Popa

**Affiliations:** 1 Department of Obstetrics and Gynecology, Elena Doamna Obstetrics and Gynecology University Hospital, “Grigore T. Popa” University of Medicine and Pharmacy, 700115 Iasi, Romania; sargu_gabriela@yahoo.com (G.D.S.); tudorandreib@gmail.com (T.B.); akad.mona@yahoo.com (M.A.); ioana-m-pavaleanu@umfiasi.ro (I.P.); 2 Department of Radiology, Elena Doamna Obstetrics and Gynecology University Hospital, “Grigore T. Popa” University of Medicine and Pharmacy, 700115 Iasi, Romania; 3 Department of Biochemistry, “Grigore T. Popa” University of Medicine and Pharmacy, 700115 Iasi, Romania; cristiana.filip@umfiasi.ro; 4 Faculty of Medicine and Biological Sciences, University Stefan cel Mare of Suceava, 720229 Suceava, Romania; andrei.cucu@usm.ro; 5 Department of Cardiology, Sf. Spiridon Hospital, 700111 Iași, Romania; amelian.bobu@gmail.com; 6 Department of Morphophunctional Sciences, “Grigore T. Popa” University of Medicine and Pharmacy, 700115 Iasi, Romania; laura_knieling@yahoo.com; 7 Department of Pharmacological Physics, “Grigore T. Popa” University of Medicine and Pharmacy, 700115 Iasi, Romania; diana.lacatusu@umfiasi.ro; 8 Department of Neurology, “Grigore T. Popa” University of Medicine and Pharmacy, 700115 Iasi, Romania; mg-rom-31105@students.umfiasi.ro; 9 Department of Vascular Surgery, “Grigore T. Popa” University of Medicine and Pharmacy, 700115 Iasi, Romania; radu.popa@umfiasi.ro

**Keywords:** embolic stroke of undetermined source, cryptogenic stroke, TOAST classification, secondary stroke prevention, atrial fibrillation, patent foramen ovale, anticoagulation, atrial cardiopathy, stroke recurrence, diagnostic workup

## Abstract

Embolic stroke of undetermined source (ESUS) was proposed in 2014 as a clinical category to subgroup non-lacunar cryptogenic ischemic strokes that appear embolic but lack an identifiable cause despite thorough investigation. The initial hypothesis was that anticoagulation might offer superior secondary prevention compared to antiplatelet therapy, prompting several large clinical trials. This review synthesizes current knowledge on ESUS. ESUS represents about 17% of ischemic strokes and often affects younger patients with fewer traditional risk factors. Although these patients lack major cardioembolic sources (e.g., atrial fibrillation) or significant arterial stenosis, many have covert embolic substrates. Major trials—NAVIGATE ESUS, RE-SPECT ESUS, and the atrial cardiopathy-focused ARCADIA—found no benefit of anticoagulants over aspirin, challenging the original ESUS framework. These results highlight the heterogeneity within ESUS and underscore the need for individualized diagnostic and therapeutic strategies.

## 1. Introduction

Ischemic stroke is a heterogeneous condition typically classified by etiology, as outlined in the Trial of Org 10172 in Acute Stroke Treatment (TOAST) system. Cryptogenic stroke diagnosis of exclusion accounts for approximately 20–30% of ischemic strokes, even after standard evaluation with brain imaging, vascular studies, cardiac workup, and laboratory testing [1,2,3]. Contemporary guidelines now recommend a more extensive diagnostic approach, including arterial imaging, echocardiography, and prolonged cardiac monitoring [3].

To refine classification within the cryptogenic category, the concept of Embolic Stroke of Undetermined Source (ESUS) was introduced in 2014 [2,4]. ESUS refers to non-lacunar ischemic strokes of presumed embolic origin in patients without identifiable high-risk cardioembolic sources or significant arterial stenosis. This concept was developed to standardize patient selection in clinical trials and to assess whether empirical anticoagulation could improve outcomes in this subgroup.

This review synthesizes recent evidence on ESUS, covering its definition and diagnostic criteria, epidemiology, and current strategies for evaluation and secondary prevention. It also reviews findings from key trials (NAVIGATE ESUS, RE-SPECT ESUS, and the 2024 ARCADIA trial), addresses ongoing controversies, such as management of patent foramen ovale and atrial cardiopathy, and explores emerging directions in the field. Our review integrates underrepresented global cohort data and critically evaluates limitations in the ESUS framework, offering a broader perspective than prior literature.

### Unique Contributions and Novelty of This Review

This review distinguishes itself from prior literature in several key ways. First, it integrates the most recent clinical trial data, including results from the 2024 ARCADIA and ATTICUS trials, providing an updated synthesis not available in earlier reviews. Second, it includes underrepresented global data, such as findings from Latin American and Asian ESUS cohorts, expanding the generalizability of insights beyond Western populations. Third, it provides a critical appraisal of the ESUS construct itself, emphasizing its evolving utility and limitations in guiding therapy, particularly in light of recent null results from large randomized trials. Finally, this review incorporates emerging technologies in diagnosis, such as wearable cardiac monitoring and machine learning-assisted risk stratification, offering a forward-looking perspective on personalized care in ESUS management. These elements collectively offer a more contemporary and globally relevant evaluation of the ESUS paradigm than existing reviews.

## 2. Materials and Methods

This review was conducted as a structured narrative review, aiming to synthesize the most relevant and high-quality literature on embolic stroke of undetermined source (ESUS) from recent years. Although not a systematic review, we followed a defined protocol to ensure transparency and reproducibility of the evidence selection process.

### 2.1. Databases and Search Strategy

We searched three major electronic databases: PubMed, Embase, and Scopus. The searches were performed in January 2025 and covered literature published from January 2018 to December 2024. Earlier foundational studies were included selectively for historical context and conceptual clarity. The search strategy utilized combinations of medical subject headings (MeSH) and keywords, including but not limited to: “embolic stroke of undetermined source”; “ESUS”; “cryptogenic stroke”; “stroke of unknown origin”; “ESUS AND prevalence”; “ESUS AND anticoagulation”; “cryptogenic stroke AND trial”; “NAVIGATE ESUS”; “RE-SPECT ESUS”; and “ARCADIA trial”.

Boolean operators (“AND”, “OR”) were applied to broaden or refine the search results. Filters were set to retrieve English language articles, published in peer-reviewed journals, and with full-text availability.

### 2.2. Inclusion and Exclusion Criteria

Inclusion criteria: Articles addressing the definition, epidemiology, risk factors, diagnostic workup, or management of ESUS; clinical trials, systematic reviews, meta-analyses, large cohort studies, and consensus guidelines; studies comparing anticoagulation vs. antiplatelet therapy in ESUS; and papers focusing on ESUS subgroups (e.g., atrial cardiopathy, PFO-associated stroke)

Exclusion criteria: studies focused exclusively on pediatric or non-ESUS cryptogenic stroke; case reports or single-center studies with limited generalizability; abstracts without accompanying full-text publications; and non-English articles.

### 2.3. Study Selection and Data Extraction

Initial search results were screened by title and abstract. Full texts of potentially relevant articles were then reviewed independently by two authors to determine final inclusion. Data were extracted and synthesized narratively, focusing on population characteristics, study design, primary outcomes, and implications for clinical practice.

## 3. Definition and Diagnostic Criteria of ESUS

**Figure 1:**
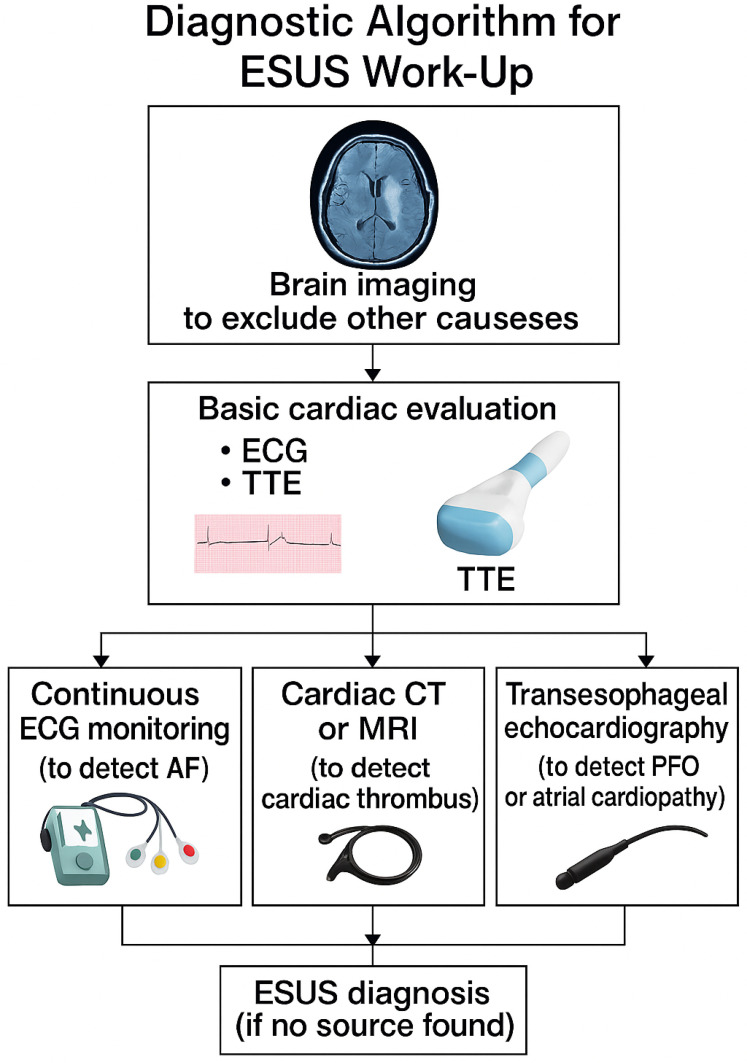
Diagnostic algorithm for ESUS workup.

Embolic stroke of undetermined source (ESUS) describes non-lacunar ischemic strokes with imaging features suggestive of embolism, occurring in patients without major-risk cardioembolic sources or significant large-artery atherosclerosis [2]. ESUS is a subset, but not a synonym of, cryptogenic stroke, requiring a relatively complete diagnostic workup to exclude identifiable etiologies (Figure 1, Table 1).

Diagnostic criteria proposed by the ESUS International Working Group [2] include:

- Absence of ≥50% stenosis in arteries supplying the affected territory
- No major cardioembolic source (e.g., atrial fibrillation, intracardiac thrombus)
- Non-lacunar infarct on imaging
- Completion of minimum diagnostic tests: brain and vascular imaging, ≥24 h ECG monitoring, transthoracic echocardiography, and basic laboratory evaluation. Transesophageal echocardiography and thrombophilia testing may be added based on clinical suspicion.

The ESUS framework aims to identify a population likely to benefit from targeted secondary prevention strategies, particularly anticoagulation, based on presumed embolic pathophysiology.

## 4. ESUS in the Context of Ischemic Stroke Classification (Relation to TOAST)

In the traditional TOAST classification, cryptogenic stroke serves as a broad category for ischemic strokes of an unknown cause [1]. This includes cases with incomplete evaluations, multiple competing causes, or no clear etiology found. As a result, the cryptogenic label is heterogeneous—encompassing everything from lacunar strokes with unconfirmed cause to possible embolic events without documented atrial fibrillation (AF) [1,4].

The introduction of ESUS aimed to define a more homogeneous subset of cryptogenic strokes by applying stricter criteria. ESUS includes non-lacunar infarcts with a presumed embolic pattern and requires a thorough diagnostic workup that excludes high-risk cardiac and large artery sources [2]. Patients with incomplete evaluations or multiple potential causes remain in the broader cryptogenic category under TOAST, but do not meet ESUS criteria. Likewise, classic lacunar infarcts are excluded from ESUS—even if no cause is found—due to their presumed small vessel origin.

The rationale behind ESUS was that many cryptogenic strokes have embolic features (e.g., cortical location or multiple vascular territories), suggesting occult embolism from undetected AF or structural cardiac sources. Emerging diagnostic tools, such as prolonged rhythm monitoring and advanced imaging, were increasingly uncovering covert embolic sources—strengthening the hypothesis that empiric anticoagulation might benefit these patients [2,5].

However, it is important to note that ESUS is an operational research definition rather than a distinct pathophysiologic mechanism. It remains a diagnosis of exclusion. Some critics pointed out that labeling a stroke as ESUS does not by itself reveal the cause—it merely indicates a failure to find the cause with current methods [6,7]. After the neutral results of the ESUS trials (discussed later), there has been debate about whether the ESUS construct adds value in clinical practice or if efforts should refocus on exhaustive diagnostic pursuit of specific etiologies [8]. ESUS has been widely adopted in stroke research and is useful for discussing cryptogenic strokes with presumed embolic pathways.

In summary, relative to TOAST:

Cryptogenic stroke (TOAST) = all unexplained ischemic strokes (with various reasons for being unexplained).

ESUS (2014 definition) = a refined subset of cryptogenic strokes that are non-lacunar and have undergone a standardized, thorough workup excluding known major causes, implying an embolic mechanism from an undetermined source.

This distinction is critical in interpreting ESUS-specific epidemiology, trial design, and therapeutic approaches.

## 5. Prevalence and Incidence of ESUS

### 5.1. Prevalence

Multiple studies consistently found that ESUS constitutes a substantial portion of all ischemic strokes. A 2017 systematic review by Hart et al. reported that ESUS accounts for roughly one in six ischemic strokes, with reported frequencies ranging from 9% to 25% across different stroke cohorts (average ~17%) [9]. Similarly, an international multicenter registry (ESUS Global Registry) found that about 16% of recent ischemic stroke patients met ESUS criteria, and this proportion was remarkably consistent across diverse global regions (16–21% in North America, Europe, Asia, etc.) [10]. These data underscore that ESUS is not a rare entity; on the contrary, it represents a common stroke subtype worldwide [4].

It is worth noting that an additional fraction of patients (often around 10–15%) have strokes that are cryptogenic due to incomplete evaluation. For example, the ESUS Global Registry reported that another 14% of patients had an incomplete workup precluding ESUS diagnosis [10]. Many of these might have qualified as ESUS if fully evaluated. Thus, with thorough investigations becoming more routine (e.g., prolonged Holter monitoring, etc.), the proportion of strokes classified as ESUS may increase as some previously uncharacterized cryptogenic strokes become appropriately categorized.

Table 2 summarizes prevalence findings from key studies. Notably, the prevalence can vary depending on the setting: tertiary stroke centers that perform exhaustive diagnostic testing may report a higher proportion of ESUS (because more cryptogenic strokes are characterized as ESUS rather than left undetermined). Conversely, community settings with less extensive workups might label more strokes as generic cryptogenic (thereby potentially underestimating ESUS). Overall, however, most large series in the past decade converge on an ESUS frequency in the 15–20% range among ischemic strokes [4,9].

### 5.2. Incidence

When expressed as an incidence rate in the general population, ESUS has been estimated at roughly 1.5–2.5 per 1000 person-years in the United States [6]. This reflects the population-level occurrence of ESUS, though precise incidence is hard to pin down and depends on stroke incidence and workup patterns. Given that overall ischemic stroke incidence increases with age, and about a sixth of those are ESUS, the incidence of ESUS similarly rises with age. There is no evidence of a secular trend yet for ESUS incidence; however, improvements in diagnostic monitoring could paradoxically decrease ESUS incidence in the future by reclassifying some cases to known etiologies.

### 5.3. Recurrent Stroke Risk

ESUS patients face a meaningful risk of recurrent stroke, averaging 4–5% per year on antiplatelet therapy. Observational studies and trials such as Hart et al. (2017) [9] and NAVIGATE ESUS report recurrence rates of ~4.5–4.7% annually with aspirin [9,14]. This risk is comparable to general cryptogenic stroke populations, lower than in high-risk cardioembolic stroke (e.g., untreated AF), but higher than in lacunar stroke.

Notably, recurrent events in ESUS patients are frequently also cryptogenic. A secondary analysis of NAVIGATE ESUS showed that most recurrences remained undetermined in origin, suggesting a persistent occult mechanism [15]. These findings underscore the need for effective secondary prevention, though whether anticoagulation improves outcomes remains unresolved.

## 6. Demographic Differences in ESUS

### 6.1. Age

ESUS patients are generally younger than those with strokes due to large-artery atherosclerosis or atrial fibrillation. The ESUS Global Registry reported a mean age of 62 for ESUS vs. 68 for non-ESUS strokes [10], while Hart et al. (2017) [9] found a pooled mean age around 65 [9]. The younger profile may reflect fewer overt etiologies in middle-aged adults, whereas older patients more often have detectable AF or severe atherosclerosis [16]. Still, ESUS spans all age groups, with younger patients more likely to have PFO or thrombophilia, and older patients more likely to harbor covert AF or aortic plaque.

### 6.2. Sex

Sex distribution is approximately balanced. Hart et al. reported 58% male and 42% female in pooled ESUS data [9], and the ESUS Global Registry found no significant sex-based difference in ESUS prevalence [10]. Minor trends exist (e.g., PFO-related strokes may be more common in young men), but no strong sex predilection has been observed.

### 6.3. Geographic and Ethnic Differences

ESUS prevalence is consistent across global regions (typically 15–21%) [10]. Some studies suggest ethnic variations in underlying mechanisms—for example, South Asian ESUS patients may have earlier onset and more cardiogenic sources (e.g., rheumatic disease, early AF) [13], while Western cohorts may have more subclinical atherosclerosis. However, robust ethnic data remain limited.

### 6.4. Stroke Severity

ESUS strokes tend to be milder than large vessel or AF-related strokes. Median NIHSS scores at presentation are typically 4–5, compared to ≥8–10 in cardioembolic stroke [9,10]. This likely reflects smaller emboli or distal occlusions, although severe ESUS presentations can occur.

### 6.5. Underlying Covert Pathologies

While ESUS patients lack known AF by definition, long-term monitoring reveals paroxysmal AF in ~20–30%, particularly among older patients. PFO is more common in younger ESUS patients (typically <60), with ~9% identified in NAVIGATE ESUS [14]. Older patients are more likely to have covert aortic arch plaque (>4 mm), which may serve as an unrecognized embolic source [4].

## 7. Risk Factors and Patient Profile in ESUS

While ESUS by definition lacks an identified cause, patients often have underlying risk factors or clues that can guide suspicion towards potential embolic sources. The “risk factor profile” of ESUS can be considered on two levels: (1) traditional stroke risk factors (such as hypertension, diabetes, smoking, etc.), and (2) specific factors that might point to covert embolic sources (such as markers of atrial cardiopathy or presence of a PFO).

### 7.1. Traditional Vascular Risk Factors

Studies suggest that ESUS patients tend to have fewer or less pronounced traditional risk factors compared to other stroke subtypes. In a recent factor analysis of risk variables in ESUS versus non-ESUS strokes, ESUS patients were found to cluster less with the typical metabolic syndrome factors and more with cardiac and aortic factors [17]. Common risk factors in ESUS include:

1. Hypertension: Present in a majority of ESUS patients (~60–70%), but often not as long-standing or severe as in lacunar stroke patients [10,17]. Hypertension can cause small vessel disease, but in ESUS patients, the hypertension may contribute indirectly (e.g., to atrial enlargement or aortic stiffness) rather than directly.
2. Diabetes mellitus: Seen in roughly 20–30% of ESUS patients [10]. This is slightly lower than in large artery atherosclerotic stroke populations. Poorly controlled diabetes tends to cause microvascular disease, which would manifest as lacunar infarcts (excluded from ESUS). Thus, ESUS diabetics might be those with relatively moderate disease.
3. Smoking: Many ESUS patients have a history of smoking, though exact proportions vary by region. Smoking is a risk factor for atherosclerosis; heavy smokers might develop carotid plaques. In ESUS cohorts, smoking rates have been reported anywhere from ~20% to 40% [4,10]. If significant atherosclerosis from smoking is absent, its role might be through promoting hypercoagulability or cardiac arrhythmia triggers.
4. Dyslipidemia: Hyperlipidemia is common but typically managed—many ESUS patients are found to have elevated cholesterol or are on statins for primary prevention. Dyslipidemia contributes to atherosclerosis; hence severe dyslipidemia would lead to an identified plaque source (and not ESUS). However, moderate dyslipidemia can be present without overt plaque >50%. The risk factor profiles often show that ESUS patients resemble the general stroke population [17].
5. Prior stroke or transient ischemic attack (TIA): A history of a prior stroke or transient ischemic attack is noted in some ESUS patients (~15–20%) [10]. A prior unexplained stroke suggests an underlying persistent but undetected cause [14]. Patients with recurrent cryptogenic strokes warrant especially intensive evaluation for occult causes.

### 7.2. Potential Covert Embolic Sources and Associated Factors

This is a critical aspect of the ESUS patient profile. Although no major source is identified, many ESUS patients have subclinical conditions that are plausible embolic sources. Some of these include the following:

1. Atrial cardiopathy: Even without diagnosed atrial fibrillation (AF), patients may show signs of atrial dysfunction—referred to as atrial cardiopathy. Markers include left atrial enlargement on echocardiography, elevated NT-proBNP, abnormal P-wave indices on ECG (e.g., increased P-wave terminal force in V1), or atrial fibrosis on cardiac MRI. Studies report that 30–40% of ESUS patients exhibit one or more of these features, suggesting an underlying atrial substrate prone to thromboembolism [18]. Elevated NT-proBNP is associated with both cryptogenic stroke and future AF detection, and patients with atrial cardiopathy are typically older and may develop AF with longer monitoring.
2. Subclinical atrial fibrillation: Although AF is absent by definition in ESUS, prolonged cardiac monitoring often reveals paroxysmal AF. The CRYSTAL-AF study found AF in 12.4% of cryptogenic stroke patients at 12 months and about 30% at 3 years using an implantable loop recorder [5]. The EMBRACE trial similarly detected AF in 16% of patients with 30-day external monitors [19]. Risk factors for subclinical AF include older age, structural heart disease, and elevated NT-proBNP. In the NAVIGATE ESUS trial, only ~3% of patients had AF detected over a median 11 months of standard monitoring [20], though this likely underestimates the true prevalence due to limited follow-up duration.
3. Patent foramen ovale (PFO): PFO, a potential route for paradoxical embolism, is present in about 25% of the general population and in 40–50% of younger patients (<55) with cryptogenic stroke [21]. In ESUS, PFOs are included unless proven causative (e.g., with associated DVT). High-risk features (e.g., large shunt, atrial septal aneurysm) increase the likelihood of causality, particularly in younger patients without vascular risk factors. Trials have shown that closure of high-risk PFOs reduces recurrence in cryptogenic stroke [21,22]. In NAVIGATE ESUS, patients with PFO did not show differential benefit from rivaroxaban vs. aspirin [14], suggesting that anticoagulation is not necessarily indicated unless PFO is clearly causal.
4. Non-stenosing atherosclerotic plaques: Some ESUS patients have non-occlusive atherosclerotic plaques (<50% stenosis) in the aortic arch or carotid arteries that may still cause embolism. Aortic plaques ≥4 mm and complex carotid lesions (ulcerated or mobile) are associated with stroke risk despite falling below the threshold for large-artery categorization. These findings, more common in older individuals, smokers, and those with hyperlipidemia, may represent an arterio-embolic ESUS subgroup. Such patients may benefit more from intensive antiplatelet or statin therapy than anticoagulation [17].
5. Left ventricular (LV) dysfunction: Subtle LV abnormalities—such as wall motion deficits or mildly reduced ejection fraction—may exist in ESUS patients without meeting criteria for cardioembolic stroke. These could reflect silent myocardial infarction or undetected ventricular thrombi. Studies such as that by Perkins et al. (2022) [17] found clusters of LV dysfunction and aortic plaque, hinting at a cardioaortic embolic mechanism. These patients may have coronary disease history, but no overt cause detected during acute stroke evaluation. Advanced imaging (e.g., cardiac MRI) may reveal missed sources [17].
6. Hypercoagulability and cancer-associated stroke: While rare, prothrombotic conditions and occult cancer can present as ESUS. Malignancy may cause embolic-appearing strokes via nonbacterial thrombotic endocarditis or coagulation disturbances. If cancer is already known, the stroke is often classified as cancer-associated. However, undiagnosed malignancy may be present in ESUS patients, particularly older individuals. Up to 5–10% of cryptogenic strokes in elderly patients reveal cancer within a year [23]. Clues include high D-dimer levels, weight loss, or systemic symptoms. Treatment often involves anticoagulation and managing the underlying cancer.

### 7.3. Predictors of Recurrence

History of prior stroke is the strongest predictor of another stroke in ESUS [24]. Other predictors include age (older ESUS patients have higher recurrence risk, presumably because they are more likely to have an underlying AF that will cause another stroke) and the presence of covert atrial cardiopathy markers [4]. Identifying these risk factors is important because they might guide more aggressive monitoring or empiric therapy.

In conclusion, ESUS patients often straddle an intermediate risk factor profile: they are not the “low-risk, no-risk-factor” population (since those rarely have stroke at all), but they also are not the “heavy vascular risk” group where a cause leaps out. Instead, they frequently have subtle or emerging risk factors pointing to occult cardioembolism or arterioembolism. This nuanced profile is exactly what makes managing ESUS challenging—one has to anticipate and search for a hidden cause that is not immediately apparent from standard risk factors alone.

## 8. Current Controversies and Diagnostic Strategies

The concept of ESUS generated considerable debate, particularly after clinical trials failed to show the expected benefit of anticoagulation. Several controversies surround the identification and management of ESUS:

1. Utility of the ESUS construct: A major controversy is whether labeling a stroke as ESUS meaningfully influences clinical decision-making. Proponents argue it emphasizes an embolic mechanism and promotes standardized workup and consideration of anticoagulation. Critics counter that the label does not change therapy—especially after trials failed to show benefit from empiric anticoagulation—and may discourage continued diagnostic efforts [6,8]. They highlight that ESUS is not fundamentally distinct from cryptogenic stroke, yet was widely adopted before evidence of clinical benefit. Many advocate instead for deeper diagnostics—e.g., prolonged monitoring, plaque imaging, or thrombophilia screening—to reclassify ESUS cases into specific stroke subtypes [12]. This sparked debate between empiric treatment vs. extended diagnostic strategies.
2. Extent of diagnostic workup: Another area of debate is how far to go in evaluating a stroke before labeling it ESUS. While a minimum workup is defined, many experts recommend extended cardiac monitoring (≥30 days) in all patients with embolic-appearing cryptogenic strokes [3,5,19] due to the high yield of detecting paroxysmal AF. Transesophageal echocardiography (TEE), though not required by ESUS criteria, may uncover relevant findings such as PFO or complex aortic plaque. Likewise, cancer screening (e.g., CT, tumor markers) may be warranted in older patients with elevated D-dimer or other red flags. Genetic testing for thrombophilia is not routinely advised, but may be appropriate in younger patients with suggestive histories. Ultimately, the depth of evaluation remains a judgment call, with some clinicians stopping once ESUS criteria are met, and others pursuing exhaustive workup.
3. Management of PFO in ESUS: The presence of a PFO in ESUS patients is a gray zone. Technically, patients with PFO but no DVT or alternate cause still qualify as ESUS. However, with the advent of PFO closure trials showing benefit in select patients, many clinicians now treat these as paradoxical embolisms, especially in younger patients with high-risk PFO features [21,22]. Patients <60 with isolated PFOs are often reclassified and considered for closure. In contrast, small or incidental PFOs in older adults remain controversial. Although ESUS definitions historically included PFO, evolving evidence is reshaping clinical management.
4. Role of anticoagulation vs. antiplatelet: Initial ESUS trials (NAVIGATE ESUS, RE-SPECT ESUS) hypothesized that empiric anticoagulation would reduce recurrence. However, both trials found no benefit of DOACs over aspirin and highlighted bleeding risks [20,25]. This outcome spurred debate about whether the failure was due to ineffective treatment or the inclusion of a heterogeneous population. Subgroup analyses suggested possible benefit in specific populations—e.g., patients ≥75 years or those with LV dysfunction (EF < 50%) [26,27]. These findings support a move toward more individualized strategies, guided by markers such as atrial cardiopathy. While guidelines now generally favor antiplatelets for ESUS absent another indication [3]. The controversy reflects the tension between generalized treatment vs. personalized care.
5. ESUS vs. further subdivision: Another debate is whether the umbrella of ESUS is too broad. Alternative approaches, such as the A-S-C-O-D system, grade potential etiologies rather than assign a binary label [28]. For example, a patient might have minor atrial cardiopathy and non-stenotic aortic plaque—both possible sources. This raises the question: should treatment be tailored based on these findings (e.g., anticoagulate atrial ESUS, intensify statins for arterial ESUS)? Since the original ESUS trials did not stratify by these features, benefits may have been diluted. Recent and ongoing trials now target narrower phenotypes. The debate continues as to whether ESUS should remain a clinical category or evolve into a transitional research term, with the ultimate goal being precise etiologic diagnosis [4,29].

Table 3 compares major international guideline recommendations for the management of ESUS, highlighting areas of consensus and divergence between the AHA/ASA 2021 and ESO 2022.

### Diagnostic Strategies

To maximize the chances of finding an underlying cause and thereby exiting the ESUS category to a more definitive one, the following strategies are considered:

1. Prolonged cardiac monitoring: As mentioned, detecting occult atrial fibrillation can be game-changing (because it shifts management to anticoagulation unequivocally). Current best practice for ESUS (cryptogenic stroke) is to perform at least several weeks of monitoring if initial telemetry and Holter are unrevealing [3]. This can be achieved via a 30-day event monitor, multiple shorter Holters, or an implantable loop recorder depending on patient risk and resources. Many stroke centers now place implantable cardiac monitors in patients with cryptogenic stroke who are at moderate-to-high risk for AF (for instance, age >55 or evidence of atrial cardiopathy). As an example, a CRYSTAL-AF trial showed the benefit of an implantable monitor, and thus in a patient who is willing and where AF suspicion is high, this is a reasonable step [5]. Even the ARCADIA trial, which required no known AF at baseline, found that about 25% of participants were diagnosed with AF during follow-up [30], underscoring how common subclinical AF is.
2. Imaging for structural cardiac sources: Transthoracic echocardiography (TTE) is routine, but transesophageal echocardiography (TEE) is often more sensitive for detecting things such as PFO (via bubble study), atrial septal aneurysm, valvular strands, or aortic arch atheroma [5]. In an ESUS workup, a TEE can be very revealing and is recommended especially in patients <60 or in older patients if no other clue is found [2]. Likewise, if TEE confirms a PFO with a large shunt, that patient might be referred for PFO closure [5]. Thus, TEE plays a diagnostic and potentially therapeutic role. Additionally, if cardiomyopathy is possible, cardiac MRI provides tissue characterization (for example, late gadolinium enhancement in the left atrium might indicate fibrosis predisposing to AF) [11].
3. Vascular imaging and novel techniques: Standard vascular imaging (carotid ultrasound, CTA, or MRA) is conducted to rule out >50% stenosis. However, newer techniques, such as high-resolution MRI of vessel walls, can sometimes identify features in non-stenotic plaques (such as intra-plaque hemorrhage or ulceration) that suggest them as the culprit [12]. Some research imaging can show plaque inflammation (using PET scans) or detect microembolic signals on transcranial Doppler, indicating an active embolic source in arteries [12]. These are not yet routine, but they represent how technology might pin down an arterial source in ESUS.
4. Laboratory workup: All ESUS patients should have basic labs for hypercoagulability and other clues: e.g., antiphospholipid antibody panel in younger patients or if there is any autoimmune clue, coagulation factors, platelet count (for rare disorders), inflammatory markers (for vasculitis), or cancer screening labs if indicated (such as CEA, PSA, etc., not routinely conducted without suspicion). A particularly useful test is D-dimer; an elevated D-dimer in cryptogenic stroke has been associated with cancer-related strokes or prothrombotic states. Extremely high D-dimer might prompt screening for malignancy or deep vein thrombosis (with the idea that maybe a venous clot crossed a PFO) [23]. ESUS patients usually do not show gross lab abnormalities; if they do (such as very high antiphospholipid titers), then one would reclassify the stroke cause (e.g., “antiphospholipid syndrome” rather than ESUS) [4].
5. Neuroimaging patterns: Radiologically, clinicians examine the stroke pattern on MRI to glean insights. For instance, an infarct in multiple vascular territories (different sides or anterior and posterior circulation simultaneously) strongly suggests an embolic shower, often cardiac [2]. ESUS patients with such patterns might be prioritized for arrhythmia monitoring. Conversely, a single small cortical infarct might raise consideration of local plaque. Diffusion-weighted MRI “lesion mapping” is being studied to differentiate likely cardioembolic vs. artery-to-artery mechanisms. Although not definitive, this is part of the art of ESUS evaluation [4] (Table 4).

## 9. Importance of Accurate Identification of Stroke Etiology

**Figure 2:**
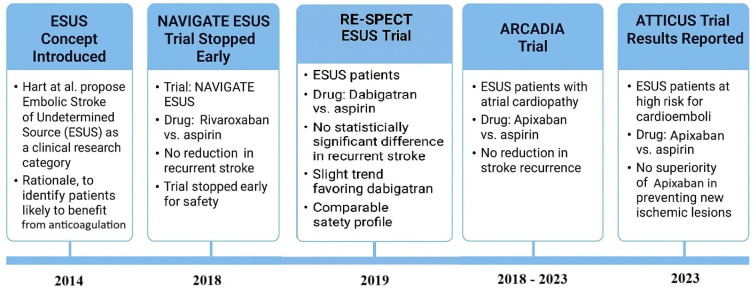
Timeline of ESUS trials.

Accurate identification of the stroke mechanism in ESUS patients is critically important because it directly influences secondary prevention strategies and outcomes. Several points highlight this importance:

1. Therapeutic implications: Stroke recurrence prevention depends on identifying the correct mechanism. If atrial fibrillation (AF) is discovered, oral anticoagulation reduces recurrence risk by about two-thirds compared to antiplatelets. Similarly, detection of large artery disease (e.g., carotid stenosis) can lead to surgical intervention, and identification of a PFO with venous thrombosis may prompt closure or anticoagulation. Cancer-related stroke requires treatment of the underlying malignancy and anticoagulation. In each case, missing the diagnosis and defaulting to aspirin alone, as in ESUS management, may expose patients to unnecessary recurrence risk.

2. Failed trials and lessons learned: The neutral results of the ESUS anticoagulation trials (NAVIGATE ESUS and RE-SPECT ESUS) underscore the heterogeneity of ESUS and reinforce why accurate identification is needed (Figure 2). These trials effectively treated ESUS as a single entity that could be managed en bloc with one therapy. NAVIGATE ESUS (rivaroxaban 15 mg daily vs. aspirin 100 mg) included 7213 patients and found no reduction in recurrent stroke with rivaroxaban (annual recurrence ~4.7% in both groups), but a higher rate of major bleeding (1.8% vs. 0.7% per year) [20]. RE-SPECT ESUS (dabigatran 150/110 mg BID vs. aspirin) with 5390 patients similarly showed no significant difference in recurrent stroke (4.1%/yr dabigatran vs. 4.8%/yr aspirin, HR 0.85, and *p* = 0.10) and no significant major bleed difference (though more minor bleeds with dabigatran) [25]. These failed trials suggest that treating all ESUSs with a single approach (anticoagulation) was not broadly effective. The trials therefore taught that precision is needed: giving everyone a “cardioembolic” treatment without confirming a cardioembolic source failed to improve outcomes. In hindsight, this reinforces the importance of trying to stratify ESUS patients. For instance, if one could accurately identify those with occult AF, one would treat them with anticoagulant (and indeed, presumably those patients in the trial did benefit from rivaroxaban/dabigatran, but they were diluted by others who did not benefit). Conversely, those with arteriogenic emboli might have been harmed by not receiving intensive antiplatelet therapy (or by obtaining an anticoagulant that does not stabilize plaques as well as dual antiplatelets might). In fact, secondary analyses attempted to parse out subgroups. They found no subgroup with a dramatic benefit, but hinted that older patients (who likely have atrial sources) might benefit more [26], whereas patients with evidence of non-atrial sources did not. These analyses emphasize that the one-size-fits-all approach in ESUS is flawed, and accurate etiological identification or at least risk stratification is essential to personalize therapy (Table 5).

3. Recent developments in ESUS research (2025): Recent combined data from the NOAH-AFNET 6 and ARTESiA trials indicate that in patients with device-detected (subclinical) AF, oral anticoagulants (edoxaban or apixaban) significantly reduce the risk of ischemic stroke by roughly 30% compared to antiplatelet therapy. However, this benefit comes at the cost of increased major bleeding (~60% relative increase) [30]. These 2024/25 findings confirm a trade-off: anticoagulation can prevent strokes in ESUS-related subclinical AF, but with higher bleeding risk [30].

A prespecified subgroup analysis (2024) pooled NOAH-AFNET 6/ARTESiA results to examine patients with vascular disease. ESUS patients with concomitant vascular disease appeared to derive greater stroke prevention benefit from anticoagulation (incidence rate ratio ~0.75 for recurrent stroke/systemic embolism with DOAC vs. aspirin) than patients without vascular disease (no significant risk reduction, IRR ~1.0) [30]. Major bleeding risk was elevated with DOAC in both subgroups, though the absolute bleeding difference was somewhat less pronounced in the vascular disease group [30]. This analysis suggests that vascular comorbidities may identify ESUS patients more likely to benefit from anticoagulation, underscoring the need for individualized risk-benefit assessment.

The European Stroke Organisation released updated guidelines (late 2024) for cryptogenic stroke with patent foramen ovale. They strongly recommend PFO closure (plus antiplatelet therapy) for secondary prevention in 18–60-year-old patients with likely PFO-attributable stroke, especially those with high-risk PFO features (e.g., large shunt and atrial septal aneurysm) [31]. The ESO advises against routine long-term anticoagulation in PFO-associated strokes unless another indication exists [31]. Notably, evidence was deemed insufficient to recommend PFO closure in patients >60 years old, and the guideline highlights uncertainty about the benefit of routine prolonged cardiac monitoring (e.g., implantable loop recorders) after PFO closure [31].

New studies are exploring wearable technologies for atrial fibrillation detection in ESUS patients as a strategy to guide prevention. A 2024 systematic review reported that extended ECG monitoring with wearable devices (patches, smartwatches, etc.) can detect previously unknown paroxysmal AF in approximately 10–20% of patients after cryptogenic stroke [32]. Importantly, in the few studies directly comparing wearables to standard monitoring (Holter or implantable loop recorders), no significant difference in AF detection yield was found [32]. While wearables show promise for convenient, longer-term rhythm surveillance, more data are needed to determine if they improve stroke risk stratification or outcomes in ESUS beyond traditional monitoring [32].

4. Preventing recurrence and reducing mortality: Strokes of an undetermined source can recur, and recurrent strokes may be more severe, or even fatal. If the underlying cause is discovered after a first ESUS stroke, one might then institute the proper treatment, but by then, the patient may have suffered significant disability. Therefore, early identification is key to prevent that second hit. Moreover, certain causes, such as AF or large aortic plaques, also carry risk of systemic emboli (to other organs) or overall higher mortality.

5. Resource allocation: There is an economic aspect—advanced diagnostics (such as long-term monitors) cost money. However, these resources are justified if they lead to a change in management that prevents a costly stroke. The cost of one severe stroke (hospital care, rehab, and lost productivity) far outweighs the cost of an implantable loop recorder, for instance. Therefore, accurately finding a cause is cost-effective in the long run if it guides stroke-specific interventions.

6. Patient confidence and adherence: Providing a clear explanation for the stroke mechanism can improve patient understanding and adherence. Patients are more likely to comply with therapy when they understand its rationale (e.g., “You have AF, and this anticoagulant prevents future strokes”), as opposed to vague guidance under the ESUS label, which may undermine trust or motivation.

7. Research and future therapies: Accurate phenotyping of ESUS patients also benefits the research community. If we can better categorize ESUS patients by the underlying mechanism, future trials can target those groups appropriately. The failure of broad ESUS trials suggests that future studies need to be more refined. Already, trials such as ARCADIA (apixaban vs. aspirin in ESUS patients with atrial cardiopathy) have taken that next step [33]. It is important to recognize that the ARCADIA trial was terminated early for futility, which limits the strength of its conclusions. The neutral outcome may reflect limitations in the selected markers of atrial cardiopathy rather than a true absence of benefit from anticoagulation. As such, ongoing research into alternative or more refined biomarkers of atrial dysfunction may yet identify subgroups of ESUS patients who could derive meaningful benefit from targeted anticoagulant therapy. Similarly, other ongoing studies are looking at dual antiplatelet vs. single antiplatelet in certain cryptogenic strokes, etc. The more accurately we identify subpopulations, the more likely we are to find beneficial treatments for them.

## 10. Future Directions and Innovations

Looking ahead, the landscape of ESUS is evolving. The lessons learned and ongoing research are guiding several future directions aimed at better detection of occult causes and improved prevention of recurrent stroke in this population.

### 10.1. Personalized Medicine and Subgroup Trials

The future likely lies in precision medicine for ESUS. Broad strokes did not work, so researchers are parsing subgroups. There is interest in reanalyzing existing trial data (NAVIGATE, RE-SPECT, etc.) using machine learning to identify clusters of patients who benefited or were harmed from anticoagulation. For instance, combining age, echo findings, biomarker levels, etc., to predict outcome differences. If such patterns emerge, they could be tested prospectively. In fact, one trial, NAVIGATE ESUS, had a subgroup analysis of aspirin vs. rivaroxaban in patients with patent foramen ovale (PFO), which did not show a difference [34], and similarly for those with left ventricular dysfunction. However, these subgroups were not chosen a priori. Future trials can enrich the population for a suspected mechanism. The ATTICUS trial, conducted in Germany, enrolled ESUS patients with elevated risk of cardiac embolism—such as those with atrial cardiopathy or left atrial enlargement—and compared apixaban to aspirin using brain MRI endpoints. The primary results, published in 2024, indicate that apixaban was not superior to aspirin in preventing new ischemic lesions. While the overall findings are neutral, ongoing secondary and subgroup analyses are investigating whether certain patient subsets may still derive benefit from anticoagulation [35].

### 10.2. Collaborative Multidisciplinary Approach

As the causes in ESUS are varied, a future ideal approach might involve a multidisciplinary team for each cryptogenic stroke patient—including a stroke neurologist, a cardiologist/electrophysiologist, perhaps a hematologist for coagulation issues, and sometimes an endocrinologist or oncologist if needed. This team can jointly evaluate the patient’s data (similar to a tumor board approach). For instance, the cardiologist might focus on subtle cardiac findings and handle the long-term monitor analysis, while the neurologist interprets imaging and coordinates care. This collaborative approach ensures all angles are considered.

In conclusion, the future of ESUS management is trending toward more granular patient characterization and tailored therapy. Continuous technological advancements in monitoring and imaging, combined with precision medicine frameworks, hold promise to further reduce the fraction of strokes that remain unexplained and to better treat those that are. As research continues, we anticipate that the term “ESUS” may eventually become less necessary—because ideally, fewer strokes will be of a truly undetermined source. Until then, ESUS serves as a reminder of the limitations of our current diagnostic reach and a call to innovate in bridging that gap, Table 6.

## 11. Limitations

### 11.1. Limitations and Controversies of the ESUS Concept

The concept of embolic stroke of undetermined source (ESUS) faced significant criticism primarily due to its heterogeneity. Although intended to identify a subgroup of cryptogenic strokes with presumed embolic origin, evidence suggests ESUS includes a diverse range of underlying mechanisms [4]. Many patients have multiple potential embolic sources, such as undiagnosed atrial fibrillation (AF), aortic arch atheroma, patent foramen ovale (PFO), or cancer-associated coagulopathy [4]. This heterogeneity, and the absence of a unifying pathophysiology, undermines the utility of ESUS in guiding therapy—exemplified by the failure of anticoagulation trials. Grouping these patients may oversimplify a complex clinical reality.

Another key issue is whether ESUS effectively identifies patients who would benefit from alternative treatments. The original premise, that many ESUS cases are covert cardioembolic strokes, has not been borne out in trials, suggesting either a flawed hypothesis or inadequacy of current anticoagulants for the true underlying causes. Critics argue the definition is too broad to guide therapy meaningfully [35]. For instance, patients with atrial cardiopathy may benefit from anticoagulation, while those with minor atherosclerosis might do better with antiplatelets and risk factor management. Trials may have been “diluted” by this heterogeneity [25], prompting calls for subclassification based on biomarkers or imaging. One approach involves identifying atrial cardiopathy to define a subgroup, targeted in the ARCADIA trial, which also failed to show benefit [33].

Diagnostic inconsistency further complicates ESUS. Although the definition requires comprehensive testing, real-world practice varies; some patients are labeled ESUS without complete evaluation, while others are reclassified after subsequent findings (e.g., AF detected months later via loop recorder). Thus, ESUS can be a transient label, sensitive to the timing and extent of diagnostics. In trials such as NAVIGATE and RE-SPECT, some patients were later found to have AF, highlighting this limitation.

Moreover, not all cryptogenic strokes qualify as ESUS. Patients with dual plausible causes (e.g., 40% carotid stenosis and PFO) are excluded under ESUS criteria but remain cryptogenic under TOAST. Similarly, small deep infarcts (lacunar strokes) are excluded despite potentially lacking an identifiable cause. This limits the ESUS framework to non-lacunar strokes and leaves other cryptogenic cases outside its scope.

Ultimately, the most significant limitation is the lack of proven therapeutic benefit. Unlike other stroke subtypes that directly inform treatment (e.g., anticoagulation for cardioembolism, endarterectomy for carotid stenosis), ESUS has not led to improved outcomes. This prompted some to question whether the concept should be revised or even retired [36]. While some advocate abandoning the ESUS label in favor of thorough diagnostic workups and standard cryptogenic stroke management, others argue that ESUS advanced research and should be refined rather than discarded [4].

### 11.2. Limitations of Major ESUS Trials

A central limitation is patient heterogeneity. NAVIGATE and RE-SPECT enrolled all ESUS patients, while ARCADIA selected a subset with atrial cardiopathy. It is plausible that certain subgroups (e.g., those with covert AF or high-risk atrial cardiopathy) could benefit from anticoagulation, but the trials were not designed or powered to detect such effects. In ARCADIA, markers such as elevated NT-proBNP or abnormal P-wave terminal force may not have been specific enough to identify truly cardioembolic strokes [33]. The neutral result suggests either insufficient enrichment or that these patients may require different therapeutic approaches.

Another limitation is that trials had relatively short follow-up (NAVIGATE median 11 months due to early stop; RE-SPECT median ~19 months) [20,24]. If some ESUS patients develop AF or other issues over time, longer follow-up might reveal differences. However, longer follow-up could equally increase bleeding risk on anticoagulants, so it is hard to argue that longer follow-up would favor DOAC, but it might provide more insight into late-emerging AF incidence in both arms.

There is also the consideration of generalizability. Trial patients were in studies with certain exclusions—for instance, NAVIGATE/RE-SPECT excluded patients with high bleeding risk, severe uncontrolled hypertension, etc. ESUS patients with those characteristics might not neatly fit the trial profiles [20,25]. Nonetheless, the trials were fairly inclusive across ages and regions, making them reasonably generalizable to typical ESUS patients.

In NAVIGATE, the stroke recurrence rate on both aspirin and rivaroxaban was ~4.8% annually, suggesting rivaroxaban was no better than aspirin [20]. In RE-SPECT, dabigatran showed a non-significant trend toward benefit (HR 0.85; *p* = 0.10), though not reaching statistical significance [25]. These results raise the question of whether the trials were underpowered to detect modest effects. Still, the consistent neutral outcomes across trials lean toward a lack of meaningful benefit in unselected ESUS populations.

Rivaroxaban was associated with a higher rate of major bleeding compared to aspirin (1.8% vs. 0.7% per year) in NAVIGATE [20], while dabigatran had a more favorable bleeding profile in RE-SPECT [25], likely due to dose adjustment (110 mg in elderly). Apixaban in ARCADIA did not show excess bleeding. These differences underscore that not all DOACs carry the same risk and highlight the need to match the drug to the patient population. Some proposed testing apixaban in broader ESUS groups, but enthusiasm is limited post-ARCADIA [33].

The ATTICUS trial had several important limitations. It was terminated early for futility, reducing its power to detect modest differences. The primary outcome, MRI-detected ischemic lesions, may not reflect clinically meaningful events. The relatively small sample size and broad inclusion criteria introduced heterogeneity that could have diluted potential treatment effects. Additionally, a significant number of patients developed atrial fibrillation during follow-up, leading to crossover from aspirin to apixaban, which may have confounded outcomes [37].

As with most clinical trials, high adherence is assumed, but in real-world practice, ESUS patients may not take medications reliably. Additionally, if minor strokes or TIAs were missed, outcomes could have been affected. Nevertheless, major stroke trials generally have robust event detection protocols.

In summation, the trials did not show overall benefit, but some subgroups, older patients, and those with atrial cardiopathy or vascular comorbidities might still benefit. Their limitations reinforce a central controversy: ESUS is an appealing idea, but implementing a universal treatment for it failed, indicating we need a more nuanced approach. The concept of ESUS remains useful in research as a framework, but clinicians must be aware of its limitations. It is essentially an interim diagnosis pending further etiological discovery, and one should continuously seek hidden causes even after labeling a stroke ESUS. The current evidence does not support a change in secondary prevention based solely on the ESUS label (beyond what is conducted for cryptogenic stroke in general).

### 11.3. Limitations of This Review

As a narrative (non-systematic) review, our article has inherent limitations. First, we did not perform a formal systematic review with meta-analysis; instead, we qualitatively selected and synthesized literature. This approach introduces a degree of selection bias, as the choice of included studies and sources was based on the authors’ judgment. We attempted to mitigate this by using broad search criteria and including multiple high-quality studies, but we cannot guarantee that every relevant publication was captured. There is also a possibility of publication bias influencing the literature available—positive or prominent findings tend to be published and cited more, whereas negative or inconclusive studies might be under-represented. We included the major negative trials and prominent cohort studies to provide balance. No formal risk of bias assessment tool (e.g., ROBIS or the Newcastle–Ottawa Scale) was applied in this review; this represents a methodological limitation and may affect the objectivity of study appraisal. Another limitation is that our review emphasizes studies from roughly the past 5 to 7 years; while this ensures currency, it may underrepresent older data that could still be relevant.

Additionally, but we did not adhere to PRISMA guidelines or provide a flow diagram of the study selection. Therefore, our process is not fully reproducible as a systematic review. This narrative synthesis also did not formally grade the evidence quality or risk of bias of individual studies. Instead, we prioritized RCTs, meta-analyses, and consensus statements assuming their high impact, but we did not exclude all lower-level evidence. The reader should be aware that some conclusions are drawn from observational studies or expert consensus rather than strictly RCT evidence (for instance, discussions on risk factors and mechanism are partly based on cohort analyses).

Finally, the field of ESUS is rapidly evolving. New evidence (such as ongoing trials of advanced cardiac monitoring or novel therapies) may emerge soon after this writing. The conclusions drawn here are based on the evidence up to early 2025. Particularly, if future trials identify a subgroup of ESUS benefitting from a specific intervention, some of our statements (such as “anticoagulation has no proven benefit in ESUS”) would need updating. We aimed to highlight the most enduring concepts, but readers should consult the latest guidelines and studies for up-to-date practice changes. In summary, while this review provides a comprehensive overview, it should be interpreted in light of its narrative nature and the aforementioned limitations.

## 12. Conclusions

Embolic stroke of undetermined source is a construct born out of the recognition that a significant proportion of ischemic strokes lack an identifiable cause despite extensive evaluation. Over the past decade, the ESUS concept sharpened the focus on cryptogenic strokes that are likely embolic, leading to a wave of research to uncover hidden etiologies and test new preventative strategies. We have seen that ESUS patients, who make up roughly 1 in 6 ischemic strokes, tend to be younger and have milder strokes, yet carry a substantial risk of recurrence that mandates effective secondary prevention. The initial hope that empiric anticoagulation would dramatically improve outcomes in ESUS was not borne out by large trials, reflecting the heterogeneity within this category. These neutral trial results prompted a re-examination of ESUS as a unified entity and underscore the importance of drilling down to the root cause in each patient whenever possible.

Moving forward, the management of ESUS is poised to become more personalized. Prolonged cardiac monitoring is increasingly uncovering covert atrial fibrillation and will likely become standard for most patients with cryptogenic stroke. Advances in imaging and biomarker science are gradually enabling clinicians to detect subtle clues—whether an enlarged left atrium, a complex aortic plaque, or a PFO—that can tip the scales toward specific interventions (be it anticoagulation, intensive antiplatelet therapy, PFO closure, or others). The notion of “one treatment fits all” for ESUS has given way to a precision-medicine approach where therapy is guided by the individual patient’s risk factor profile and suspected mechanism. In essence, the field is transitioning from treating “ESUS” to treating the underlying causes that hide within ESUS.

The ESUS construct has, nonetheless, been valuable in rallying attention and research. It led to standardized criteria and global collaboration in studies, and from those efforts, we learned a great deal—not least that we must respect the diversity of causes in this population. Future research is actively exploring refined stratifications.

The approach to an ESUS patient should be twofold: implement the best available secondary prevention (which for now is usually antiplatelet therapy and risk factor modification, in the absence of a specific indication for anticoagulation) and simultaneously pursue ongoing surveillance for an elusive cause (especially atrial fibrillation). A high level of vigilance in follow-up is warranted, as causes can declare themselves over time. Multidisciplinary collaboration and patient engagement in the process are essential, given that management may pivot if and when a cause is identified.

In conclusion, while an embolic stroke of an undetermined source by definition lacks a clearly identifiable etiology at the outset, it should never be regarded as a final diagnosis. Rather, it is a starting point for an investigative journey. The ultimate success of the ESUS paradigm will be measured not by how many patients we label as ESUS, but by how many we can graduate from that label to a more definite diagnosis with an optimized treatment—thus improving outcomes and closing the loop on strokes once deemed “of undetermined source.”

### Key Takeaways and Implications

- ESUS remains a useful diagnostic framework that prompts thorough evaluation but should not be treated as a definitive endpoint. Clinicians must recognize its limitations and seek individualized explanations.
- Routine anticoagulation is not recommended in unselected ESUS patients based on the neutral outcomes of NAVIGATE ESUS and RE-SPECT ESUS. Treatment should default to antiplatelet therapy unless a high-risk source is later identified.
- Prolonged cardiac monitoring is essential, especially in older patients or those with signs of atrial cardiopathy, to detect covert atrial fibrillation and adjust secondary prevention accordingly.
- Future research should prioritize the following:
- Developing tools to stratify ESUS patients by a likely embolic source.
- Identifying predictive biomarkers and imaging signatures.
- Conducting trials focused on targeted subgroups (e.g., atrial cardiopathy, PFO).
- The ultimate goal is precision medicine: shifting from treating ESUS as a single entity to tailoring prevention strategies based on individualized stroke mechanisms.

## Figures and Tables

**Table 1:** Definition and required workup for cryptogenic stroke (TOAST) vs. ESUS criteria.

Classification	Definition	Required Diagnostic Workup	Reference
Cryptogenic stroke (TOAST)	Ischemic stroke of undetermined etiology: Either: - no cause found despite adequate investigation - or >1 possible cause - incomplete workup Includes lacunar or non-lacunar strokes with no identified cause.	Brain imaging (CT/MRI) Electrocardiogram (ECG) Echocardiogram Vascular imaging Relevant laboratory tests	Adams et al. (1993) [1]
Embolic stroke of undetermined source (ESUS)	Non-lacunar ischemic stroke without - proximal large-artery stenosis ≥50% - major cardioembolic source - other specific cause identified Implies an embolic mechanism is likely, though source is unknown.	Brain CT/MRI Vascular imaging of head/neck 12-lead ECG and ≥24 h cardiac monitoring (telemetry/Holter) Transthoracic echo (±TEE) Laboratory testing	(Hart et al., 2014) [2]; (Ntaios et al., 2020) [4]

Notes: AF = atrial fibrillation; ECG = electrocardiogram; CTA = CT-angiography; MRA = MR-angiography; and TEE = transesophageal echocardiography. The TOAST definition of cryptogenic stroke does not prescribe a uniform workup, whereas the ESUS criteria require a specified minimum diagnostic evaluation to confidently exclude major sources [1,2]. The AHA/ASA 2021 guidelines now endorse extended cardiac monitoring in cryptogenic stroke workup [3].

**Table 2:** Prevalence and patient characteristics of ESUS in selected studies.

Study (Year)	Population	Prevalence of ESUS	Key Characteristics of ESUS Patients	Reference
Hart et al. (2017)—Systematic Review	9 studies (multi-national) of ischemic stroke patients (2014–2016 data)	9–25% (average ~17% of ischemic strokes)	Mean age ~65 years ~42% female mostly mild strokes (mean NIHSS ~5). Average annual recurrence ~4.5% on antiplatelet therapy.	(Hart et al., 2017) [9]
Perera et al. (2016)—ESUS Global Registry	2144 stroke patients from 19 countries; hospital-based registries	16% met ESUS criteria 14% cryptogenic with incomplete workup)	Mean age 62 for ESUS vs. 68 for non-ESUS strokes 64% hypertension 25% diabetes in ESUS median NIHSS 4 (mild). 90% discharged on antiplatelet, 7% on anticoagulant.	(Perera et al., 2016) [10]
Ntaios (2020)—JACC Review (summary of prior data)	Overview of multiple cohorts	~17% of all ischemic strokes (consistent with prior findings)	ESUS patients typically younger than those with cardioembolic or atherothrombotic strokes fewer conventional risk factors lower stroke severity Annual recurrence ~4–5%.	(Ntaios, 2020) [4]
Yaghi et al. (2019)—Young ESUS Cohort (post-hoc analysis)	Young adult (≤50) stroke patients (multi-center)	Varied (ESUS was a common cryptogenic subtype in young)	Young ESUS patients had lower recurrence risk than older ESUS risk factor profile differed by age (younger had fewer vascular risk factors, more often PFO).	(Yaghi et al., 2019) * [11]
Various Regional Registries—e.g., China, India (2018–2022)	Country-specific hospital stroke registries	~15–20% (similar to global data, with some variability)	Some ethnic differences noted: e.g., South Asian ESUS patients present at a younger age and may more often have covert cardiogenic sources (Bang et al., 2014 [12]; Kaul et al., 2019 [13]) *. Overall risk factors (hypertension, etc.) present but slightly less prevalent than in non-ESUS stroke.	(Bang et al., 2014) [12] [for approach]; (Schulz, 2019) * [6]

Notes: NIHSS = National Institutes of Health Stroke Scale (stroke severity measure). * Yaghi et al. (2019) [11] and Kaul et al. (2019) [13] are illustrative; data suggest younger ESUS patients and certain ethnic groups (e.g., South Asians) may have distinctive profiles, though core criteria remain the same. These references marked with * are for context and not primary data from a single source.

**Table 3:** Comparison of ESUS recommendations: AHA/ASA 2021 vs. ESO 2022.

Category	AHA/ASA 2021 Guidelines	ESO 2022 Guidelines
Definition of ESUS	Defined as a non-lacunar infarct with no identifiable cause despite standard evaluation.	Adopts same ESUS definition but emphasizes the need for comprehensive diagnostic workup.
Initial treatment	Recommends antiplatelet therapy (aspirin) as standard secondary prevention.	Supports antiplatelet therapy; routine use of anticoagulation not recommended.
Anticoagulation in ESUS	DOACs not recommended unless specific indication (e.g., detected AF).	Recommends against routine DOAC use based on NAVIGATE/RE-SPECT outcomes.
AF monitoring	Reasonable to pursue extended monitoring (≥30 days) in cryptogenic stroke.	Advocates for extended monitoring (e.g., ILR) in selected patients, especially those ≥60 years.
Role of PFO	Considers PFO closure in patients <60 with high-risk PFO features and no other cause.	Similar stance: supports closure in young patients with high-risk PFO.
Workup recommendations	Suggests imaging, cardiac testing, and prolonged monitoring to identify stroke cause.	Emphasizes exhaustive diagnostics including TEE and MRI if suspicion for hidden cause.
Use of ESUS label	Used as an operational term, not as an endpoint. Continual diagnostic pursuit recommended.	Warns against overreliance on ESUS label; encourages refining cause where possible.

**Table 4:** Stepwise diagnostic strategies in ESUS evaluation.

Diagnostic Domain	Tool/Technique	Purpose	Key Insights/Examples
1. Cardiac monitoring	- 30-day event monitor - Implantable loop recorder	Detect occult atrial fibrillation (AF)	- CRYSTAL-AF: higher AF detection with implantables - ARCADIA: ~25% developed AF during follow-up
2. Structural cardiac imaging	- TTE (initial) - TEE (detailed, with bubble study) - Cardiac MRI	Identify PFO, atrial septal aneurysm, aortic atheroma, tumors, thrombi, cardiomyopathy	- TEE: detects high-risk aortic plaques or large PFO - Cardiac MRI: assesses fibrosis or structural heart disease
3. Vascular imaging	- Carotid US - CTA/MRA - Vessel wall MRI - PET - Transcranial Doppler	Rule out large artery disease; detect culprit non-stenotic plaques	- High-res MRI: detects intraplaque hemorrhage or ulceration - TCD: microembolic signals suggest active embolism
4. Laboratory evaluation	- Coagulation panel - D-dimer - Autoimmune markers - Cancer screening (if indicated)	Identify hypercoagulable states, malignancy, or autoimmune vasculopathy	- High D-dimer → consider cancer or paradoxical embolism - APLA panel in young stroke patients
5. Neuroimaging patterns	- MRI with DWI and vascular territory mapping	Infer embolic mechanism based on lesion distribution	- Multiple territory infarcts → likely cardioembolic - Cortical pattern → suggests embolism vs. lacunar stroke

**Table 5:** Summary of anticoagulation vs. antiplatelet trials in ESUS.

Trial	Intervention	Sample Size	Primary Outcome (Recurrent Stroke)	Major Bleeding Rate	Conclusion
NAVIGATE ESUS	Rivaroxaban vs. aspirin	7213	No significant difference 4.7%/yr vs. 4.7%/yr	Higher with rivaroxaban (1.8% vs. 0.7%)	No benefit of anticoagulation; higher bleeding risk
RE-SPECT ESUS	Dabigatran vs. aspirin	5390	No significant difference 4.1%/yr vs. 4.8%/yr HR 0.85, *p* = 0.10	Similar (dabigatran had more minor bleeds)	Trend toward benefit with dabigatran; not significant
ARCADIA	Apixaban vs. aspirin	~1100	No significant difference ~4.4%/yr in both groups	No significant difference	No benefit in atrial cardiopathy subgroup
ATTICUS	Apixaban vs. aspirin	373	No significant difference ~13.6% vs. ~16.0%	2.8% in apixaban group vs. 4.0% in aspirin group	No benefit of apixaban over aspirin; similar bleeding risk

**Table 6:** Current controversies in the management of ESUS.

Controversy	Clinical Approach	Pros	Cons
Empiric anticoagulation	Direct oral anticoagulants (DOACs) vs. antiplatelets	- Theoretical benefit in embolic mechanisms (atrial cardiopathy, occult AF) - Safe profile of DOACs in general population	- NAVIGATE ESUS and RE-SPECT ESUS: no efficacy benefit - Increased bleeding risk - Not justified without proven source
PFO closure	Percutaneous device closure in select ESUS cases	- Proven benefit in patients <60 with large shunt and no other cause - Reduces recurrent stroke risk vs. medical therapy	- Not all PFOs are pathogenic - Procedural risks (device-related complications, AF) - Requires careful selection
Atrial cardiopathy	Use of biomarkers (e.g., LA size, NT-proBNP) to guide therapy	- May identify patients at high embolic risk - Target for future trials (e.g., ARCADIA)	- No validated thresholds - Overlap with undiagnosed AF - Not yet actionable in guidelines
Duration of cardiac monitoring	24 h Holter vs. prolonged or implantable loop recorders	- Prolonged monitoring increases AF detection by up to 30% - Enables tailored anticoagulation	- Resource-intensive - May delay decision-making - Unclear how much AF burden justifies therapy
ESUS as a clinical entity	Whether ESUS should be retained as a diagnostic label	- Encourages comprehensive diagnostic workup - Useful for standardizing trial inclusion	- Concept is broad and may obscure heterogeneity - May delay pursuit of specific etiology

## Data Availability

Not applicable.

## Abbreviations

The following abbreviations are used in this manuscript:

AF	Atrial fibrillation
ECG	Electrocardiogram
CEA	Carcinoembryonic antigen
CTA	CT-angiography
DOAC	Direct oral anticoagulants
ESUS	Embolic stroke of undetermined source
FDA	Food and Drug Administration
LV	Left Ventricular
MCA	Middle cerebral artery
MeSH	Medical subject headings
MI	Myocardial infarction
NBTE	Nonbacterial thrombotic endocarditis
NIHSS	National Institutes of Health Stroke Scale
NT-proBNP	Atrial natriuretic peptides
MRA	MR-angiography
PFO	Patent foramen ovale
PSA	Prostate-specific antigen
RCT	Randomized controlled trial
TEE	Transesophageal echocardiography
TIA	Transient ischemic attack
TOAST	The Trial of Org 10172 in Acute Stroke Treatment
